# Society Meetings

**Published:** 1910-08

**Authors:** 


### SOCIETY MEETINGS.

The American Association of Obstetricians and Gynecologists will hold its twenty-third annual meeting at the Hotel Onondaga, Syracuse, N. Y., Tuesday, Wednesday and Thursday, September 20, 21 and 22, 1910, under the presidency of Dr. Aaron B. Miller, of Syracuse. Dr. Albert E. Larkin, 509 E. Genesee St., Syracuse, N. Y., is chairman of the committee of arrangements who should be addressed concerning local details, including hotel accommodations or Mr. Proctor C. Welch, manager, may be communicated with direct in relation to rooms at The Onondaga.

The following is a list of papers offered up to July 25, 1910:

1. Modern gynecology, the President's address, Aaron B. Miller, Syracuse.

2. The Semelweiss celebration at Budapest; a plea to teachers and others to do justice to Oliver Wendell Holmes, the first to point out the infective nature of puerperal fever; illustrated, Henry Schwarz, Saint Louis.

3. Puerperal wound intoxication and wound infection, Henry Schwarz, Saint Louis.

4. Pelvic reflexes, Robert T. Morris, New York.

5. Tumors of the bladder, John F. Erdmann and Joseph F. McCarthy, New York.

6. Intussusception in infants, Herman E. Hayd, Buffalo.

7. Title to be perfected, A. Vander Veer, Albany.

8. Acute surgical lesions of the upper abdomen, Charles N. Smith, Toledo.

9. Title to be perfected, Lewis C. Morris, Birmingham.

10. Intravenous injection of magnesia sulphate in bacteriemia, Raleigh R. Huggins, Pittsburg.

11. Title to be perfected, George E. Goodfellow, Guaymas, Sonora, Mexico.

12. Acute pancreatitis, John W. Poucher, Poughkeepsie.

13. Title to be perfected, Xavier O. Werder, Pittsburg.

14. Radical treatment of procidentia uteri and prolapsus recti, John B. Murphy, Chicago.

15. Appendicitis, Charles L. Bonifield, Cincinnati.

16. Fibroid uterus with special reference to degenerative changes, John B. Deaver, Philadelphia.

17. Obstruction of the bowels by malignant growths; a new method of intestinal anastomosis, Maurice I. Rosenthal, Fort Wayne.

18. The breast of the expectant mother; its care before and during the period of lactation, Francis Reder, Saint Louis.

19. Problems in uterine cancer, Walter B. Chase, Brooklyn.

20. Hospital statistics of cancer in women, K. Isadore Sanes, Pittsburg.

21. Importance of public and private hospitals in the education of young physicians and nurses, and the clinical instruction of practitioners, Joseph Price, Philadelphia.

22. Formation of a new vagina in three cases, Alexander Hugh Ferguson, Chicago.

23. Adenocarcinoma of the kidney; report of a case, J. Garland Sherrill, Louisville.

24. Advantages of suprapubic cystotomy in certain surgical conditions of the urinary bladder, J. Egerton Cannaday, Charleston, W. Va.

25. Title to be perfected, Rufus B. Hall, Cincinnati.

26. Present status of the colon tube, illustrated, H. Wellington Yates, Detroit.

27. Relation of visceral ptoses to neurasthenia, Lewis S. McMurtry, Louisville.

28. Title to be perfected, John W. Keefe, Providence.

29. Comparative merit of medical and surgical treatment in the reduction of maternal and fetal mortality in puerperal eclampsia, E. Gustav Zinke, Cincinnati.

30. Blood transfusion in pellagra, John D. S. Davis, Birmingham.

31. Cesarean section, the pregnant uterus being within an umbilical hernia, J. Henry Carstens, Detroit.

32. High operation in cesarean section, illustrated by a case report, William H. Humiston, Cleveland.

33. Torsion of the great omentum, William J. Gillette, Toledo.

34. Fibromyomata of the uterus complicating pregnancy, labor, and the puerperium, Ralph Waldo Lobenstine, New York.

35. Title to be perfected, Ernst Jonas, Saint Louis.

36. Recent advances in the technic of the radical abdominal operation for uterine cancer, Julius H. Jacobson, Toledo.

37. Results at Lebanon Hospital of deferred operations for extrauterine pregnancy, Ralph Waldo, New York.

38. Two right-sided femoral hernias coexisting in the same patient, N. Stone Scott, Cleveland.

39. Spasmodic cough of pregnancy, Douglas H. Stewart, New York.

40. Two cases of perforated gastric ulcer, Thomas B. Noble, Indianapolis.

41. Absence of pelvic organs, M. Linville, New Castle, Pa.

---

The American Public Health Association will hold its thirty-eighth annual meeting in Milwaukee, September 5 to 9, 1910, under the presidency of Dr. Charles O. Probst, Columbia. Representatives from many of the national organisations working in the interest of the public health have been invited to be present and to discuss methods for the correlation of the work of such organisations, and for coöperation with a view to increasing efficiency and economy. Sanitary engineering will occupy a conspicuous place on the program. This association is the oldest national sanitary organisation in the United States. Its membership extends over the United States, the Dominion of Canada, Mexico and Cuba. Information concerning it can be obtained by addressing Dr. William C. Woodward, Secretary, Washington, D. C.

The Fifth International Congress of Obstetrics and Gynecology will be held under the patronage of His Majesty, the Emperor of Russia, at St. Petersburg, September 22-28, 1910.

The Russian Committee on Organisation, representing every part of the Empire, has affected an organisation with Professor Dem. d'Ott, President; Prof. P. Sadovski, Secretary-General; Professor H. Zamchine, Treasurer.

The special questions to be considered by the congress are:

(1) Cesarean Section by Baum, Brandt, Doleris and Routh; (2) Non-operative Treatment of Cancer of the Uterus, by Mangiagalli, Betrix and Meyer; (3) Comparative value of the Different Procedures in the Treatment of Displacements and Deviations of the Uterus, by Schauta, Westermark and Hofmeier; (4) The Vaginal Route in Accouchement and Gynecology, by Martin, Jung, Treub and Wertheim; (5) Influence of the Nervous System in the Origin and Control of Uterine Hemorrhages, by MacNaughton-Jones and others.

In addition to the foregoing special contributions, individual essays embracing a wide range of subjects of greatest interest are already announced from over forty leading operators.

The committee for the United States consists of the following named members:

Charles A. L. Reed (chairman), Cincinnati; Robert T. Morris, New York; Howard A. Kelly, Baltimore; Lewis S. McMurtry, Louisville; Matthew D. Mann (secretary), Buffalo. Dr. Reed, 60 The Groton, Cincinnati, should be addressed concerning all details.

---

The Lake Keuka Medical and Surgical Association held its eleventh annual meeting at Grove Springs, Lake Keuka, N. Y., Thursday and Friday, July 21 and 22, 1910, under the presidency of Dr. Robert M. Elliott, of Willard. The program included papers by Drs. Roe and Ruggles of Rochester; Cook, of Canandaigua; Browning and Winfield, of Brooklyn; Tinker, of Ithaca. Discussions were made by Drs. E. H. Howard, Rochester; J. E. Walker, Hornell; Case, of Elmira, and others. Dr. Walker entertained the ladies Thursday evening by a talk on his recent tour through Spain.

---

The Medical Society of the County of Ontario at its regular meeting held at Geneva, July 15, 1910, took action favoring a law requiring the tuberculin test to be applied to cows furnishing milk for public supply. Dr. John Parmenter, president of the Geneva Public Health Association, presented the following which was adopted:

WHEREAS, Medical experience and research have proven conclusively that, while the milk of tubercular cows may be taken with more or less impunity by healthy adults, it may be and often is positively dangerous to infants and young children, there being many reported instances of infection, especially of the lymphatic and osseous systems, in early life from the digestion of tuberculous milk; therefore, be it,

*Resolved,* That the Medical Society of the County of Ontario, puts itself on record as favoring a law requiring that all cows producing milk for public consumption be submitted to the tuberculin test before such milk may be put upon the market for use.

